# *Bacillus thuringiensis* Bt_UNVM-84, a Novel Strain Showing Insecticidal Activity against *Anthonomus grandis* Boheman (Coleoptera: Curculionidae)

**DOI:** 10.3390/toxins16010004

**Published:** 2023-12-20

**Authors:** Diego Herman Sauka, Cecilia Peralta, Melisa Paula Pérez, Antonella Molla, Tadeo Fernandez-Göbel, Federico Ocampo, Leopoldo Palma

**Affiliations:** 1 Consejo Nacional de Investigaciones Científicas y Técnicas (CONICET), Ciudad Autónoma de Buenos Aires C1425FQB, Argentina; sauka.diego@inta.gob.ar (D.H.S.); cecilia.peralta@uv.es (C.P.); 2 Instituto Nacional de Tecnología Agropecuaria (INTA), Instituto de Microbiología y Zoología Agrícola (IMYZA), Hurlingham, Ciudad Autónoma de Buenos Aires 1686, Argentina; perez.melisa@inta.gob.ar; 3 Instituto Multidisciplinario de Investigación y Transferencia Agroalimentaria y Biotecnológica (IMITAB), Consejo Nacional de Investigaciones Científicas y Técnicas (CONICET), Universidad Nacional de Villa María (UNVM), Villa María 1555, Argentina; antonellabmolla@hotmail.com; 4 Laboratorio de Control Biotecnológico de Plagas, Instituto BIOTECMED, Departamento de Genética, Universitat de València, 46100 València, Spain; 5 Elytron Biotech S.A., 275 Ing. Enrique Butty Street, Ciudad Autónoma de Buenos Aires C1001, Argentina; tfernandezgobel@elytronbiotech.com (T.F.-G.); focampo@elytronbiotech.com (F.O.)

**Keywords:** *Bacillus thuringiensis*, insect pests, insecticidal proteins, Vpb1/Vpa2 proteins, Cry8 proteins, biological control, bioinsecticides

## Abstract

*Bacillus thuringiensis* is a Gram-positive bacterium known for its insecticidal proteins effective against various insect pests. However, limited strains and proteins target coleopteran pests like *Anthonomous grandis* Boheman, causing substantial economic losses in the cotton industry. This study focuses on characterizing a *Bacillus* sp. strain, isolated from Oncativo (Argentina), which exhibits ovoid to amorphous parasporal crystals and was designated Bt_UNVM-84. Its genome encodes genes for the production of two pairs of binary Vpb1/Vpa2 proteins and three Cry-like proteins showing similarity with different Cry8 proteins. Interestingly, this gene content was found to be conserved in a previously characterized Argentine isolate of *B. thuringiensis* designated INTA Fr7-4. SDS-PAGE analysis revealed a major band of 130 kDa that is proteolytically processed to an approximately 66-kDa protein fragment by trypsin. Bioassays performed with spore-crystal mixtures demonstrated an interesting insecticidal activity against the cotton boll weevil *A. grandis* neonate larvae, resulting in 91% mortality. Strain Bt_UNVM-84 is, therefore, an interesting candidate for the efficient biological control of this species, causing significant economic losses in the cotton industry in the Americas.

## 1. Introduction

*Bacillus thuringiensis* is a ubiquitous Gram-positive, sporulated bacterium well-known for its ability to produce proteins with toxic activity against insect pests, human-disease vectors (mosquitoes), and nematodes [1]. Different strains of this bacterium have been successfully used for decades in sprayable products for the biological control of insect pests (e.g., *B. thuringiensis* svar. *kurstaki*), ranging from small domestic vegetable gardens to crop fields. Moreover, the genes encoding such insecticidal proteins have been introduced into crops (Bt crops), conferring specific resistance to insect pests and promoting the reduction in the use of synthetic insecticides in integrated pest management (IPM) programs, toward sustainable agricultural practices [2]. Synthetic insecticides not only pollute the environment but also pose harm to humans, animals, and other non-target insects such as natural enemies of pests and pollinators [3]. 

*B. thuringiensis* produces its insecticidal proteins during both the vegetative growth (vegetative insecticidal proteins) and the stationary growth phases (delta-endotoxins) [4]. The vegetative insecticidal proteins include Vpb1/Vpa2 (formerly Vip1/Vip2) with activity against coleopterans, Vip (formerly Vip3) with activity against lepidopterans [5], and Vpb4 (formerly Vip4) with activity against *Diabrotica virgifera virgifera* Chevrolat (Coleoptera: Chrysomelidae) [6]. The delta-endotoxins include Cry (crystal) proteins with activity against several orders of insects and some nematodes, plus Cyt (cytolytic) proteins with activity against dipterans (mosquitoes and black flies) [7]. In addition, *B. thuringiensis* can produce Thuringiensin, also known as β-exotoxin, a non-proteinic, thermostable, and secretable secondary metabolite showing toxicity against a wide range of insects and some nematodes. Nowadays, Thuringiensin is considered an adenine nucleoside oligosaccharide analog that may interfere with RNA synthesis [8], and, as such, it has been banned from public use due to its potential toxicity against mammals and its high persistence in the environment [9]. 

However, only a few *B. thuringiensis* proteins have been described for their activity against coleopteran pests, providing a limited number of options for the biological control of these insects [10]. Therefore, finding novel strains showing coleoptericidal activity is paramount to achieving efficient crop protection against coleopteran herbivorous insects. A notable example is the cotton boll weevil, *Anthonomus grandis* Boheman (Coleoptera: Curculionidae), which is considered the most damaging pest, causing substantial economic losses to the cotton industry in the Americas [11]. Its behavior, protecting larvae from insecticides, is significant, since fertilized females lay eggs (around 200 per female) on cotton flower buds, where the main damage occurs during egg laying and larval feeding [12]. In addition, some populations in Brazil have shown resistance to certain insecticides, such as beta-cyfluthrin [13]. 

In this work, we report the molecular and insecticidal characterization of a novel *Bacillus* sp. strain isolated from Oncativo (Argentina), showing ovoid to amorphous parasporal crystals, designated as Bt_UNVM-84 strain. Its genome sequence harbors genes for the production of proteins with potential insecticidal activity against Coleoptera such as binary Vpb1/Vpa2 homolog proteins plus proteins with potential dual activity against Coleoptera and Lepidoptera, including three Cry8-like homolog proteins. Bioassays performed with spore-crystal mixtures demonstrated insecticidal activity against *A. grandis*, resulting in 91% mortality. Interestingly, a previously characterized *B. thuringiensis* strain designated INTA Fr7-4 exhibited a conserved insecticidal-gene content with a lower insecticidal activity against *A. grandis* [14]. The results provided by this study demonstrate that strain Bt_UNVM-84 emerges as a compelling candidate for the biological control of this insect species, which is causing significant economic losses in the cotton industry in the Americas. 

## 2. Results

### 2.1. Strain Isolation and Identification 

**Figure 1:**
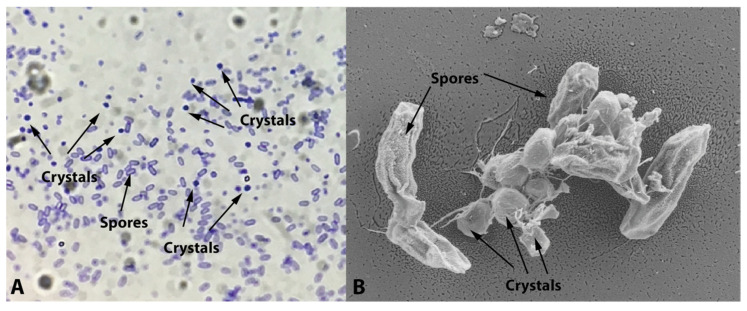
Microscopic analysis of the isolate. (**A**) Ovoid to amorphous parasporal crystals stained with Coomassie blue stain [15] and (**B**) parasporal crystals’ examination using SEM microscope.

The new *Bacillus* sp. isolate exhibited typical *B. thuringiensis* morphology in the bacterial colony (flat, dry, matt-white color with uneven borders). Under the light microscope, it also showed Coomassie-blue-stained parasporal crystals with an ovoid to amorphous shape (Figure 1A), later confirmed by SEM examination (Figure 1B). 

**Figure 2:**
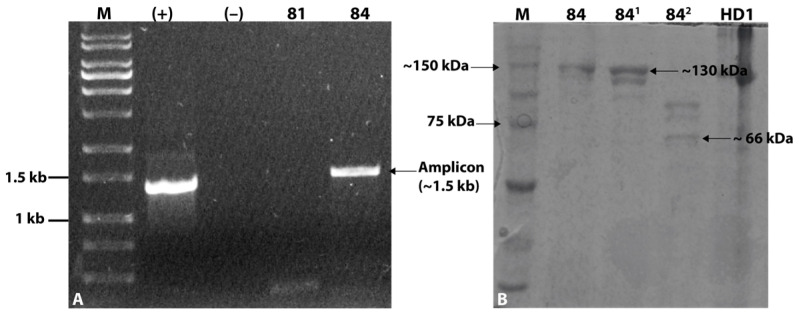
Detection of *cry* genes and proteins from the parasporal crystals. (**A**) Detection of *cry* genes by PCR with degenerate primers [16]. Amplicons were electrophoresed in 1% agarose gel: M molecular weight marker 1 kb, (+) *B. thuringiensis* strain HD1 control, (−) negative control with water; 81 is a negative control strain whereas 84 is the new isolate and (**B**) SDS-PAGE analysis: M molecular weight marker (Precision Plus Proteins Dual Color), 84 dried Bt_UNVM-84 biomass, 84^1^ solubilized Bt_UNVM-84 biomass, and 84^2^ solubilized Bt_UNVM-84 biomass digested (potentially activated) with the enzyme trypsin.

The screening of *cry* genes by PCR produced an amplicon of approximately 1.5 kb, slightly larger than the amplicon produced by *B. thuringiensis* svar. *kurstaki* HD-1 strain used as positive control (Figure 2A). SDS-PAGE analysis showed a main band of approximately 130 kDa, comparable to the size of the band from the control HD-1 strain. In addition, the band was digested by trypsin, exhibiting proteolytically digested products and a smaller band that may correspond to an activated Cry protein of approximately 66 kDa (Figure 2B). Considering these findings, the isolate was preliminary designated as *B. thuringiensis* strain Bt_UNVM-84. 

### 2.2. Genome Sequencing and Annotation

Genome sequencing produced 12,299,332 million Illumina pair-end (raw) reads, which were trimmed and assembled into 81 contig sequences, resulting in a genome size of 6,081,079 bp, with a G+C content of 34.7% with 6249 predicted protein-coding genes (CDS) and 103 RNAs. These values were consistent in both size and G + C% with other sequenced *B. thuringiensis* genomes [17,18].

**Figure 3:**
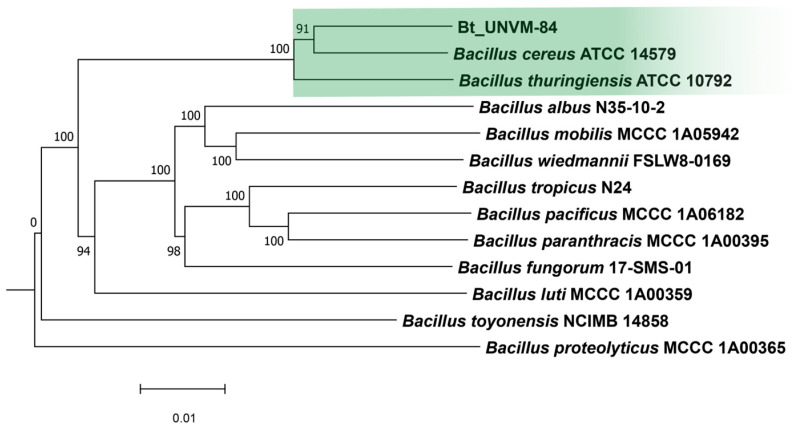
GBDP tree (whole-genome sequence-based) using TYGS server (average branch support 98.5%) [19]. Green color was used to highlight Bt_UNVM-84 strain clustered along with type strains.

Phylogenetic analysis using the Type Strain Genome Server (TYGS) showed that the new isolate branched into a unique cluster along with type strains *Bacillus cereus* ATCC 14579 and *B. thuringiensis* ATCC 10792 (Figure 3). 

In addition, mapped reads over the draft genome sequence of the related *B. thuringiensis* strain INTA Fr7-4 (Acc. no. MSFC00000000) showed 98.5% pairwise nucleotide identity covering 92.2% of the reference genomic sequence. 

The draft genome sequence from the Bt-UNVM_84 was also searched for putative insecticidal proteins and other virulence factors that may have a role in insect pathogenesis. The genome harbors seven CDSs showing significant BlastX [20] similarity with Vpa1/Vpa2 proteins and Cry8 crystal proteins.

The repertoire of insecticidal CDSs was found to be highly conserved between the INTA Fr7-4 and Bt_UNVM-84 strains. The seven homologous genes were located in the pFR260 (Acc. no. KX258624) megaplasmid harbored by strain INTA Fr7-4 strain [21]. This megaplasmid, 260,595 bp in size, encodes Cry8Qa2, Cry8Kb3, and Cry8Pa3 proteins, along with two pairs of binary proteins, namely, Vpb1Ea1/Vpa2Ah1 and Vpb1Ea2/VpaAh2. Mapping analysis using Bt_UNVM-84 Illumina reads on the pFR260 plasmid sequence showed 96.1% pairwise identity, covering 88.1% of the plasmid (used as the reference sequence). The encoded CDSs in the plasmid pFR260 showed more than 97% pairwise nucleotide identity with mapped reads from the Bt_UNVM-84 strain (Table 1). 

The Bt_UNVM-84 genome also harbors three CDSs showing 36%, 34%, and 37% pairwise amino similarity to the proteins Mpp4Aa1, Mpp46Ab1, and Xpp22Ba1, respectively. Additionally, strain INTA Fr7-4 also harbors CDS coding for Mpp4Aa1 and Xpp22Ba1, with pairwise amino similarities of 38% and 36%, respectively, but lacks the CDS encoding the Mpp46Ab1 homolog. Strain Bt_UNVM-84 also exhibited other CDSs encoding two putative chitinases and three chitin-binding proteins, whereas strain INTA Fr7-4 harbors two putative chitinases along with five chitin-binding proteins. Furthermore, the gene *thuE*, involved in the thuringiensin synthesis pathway [8,9], was not detected either by RAST server, custom BlastX analyses, or PCR amplification, following the methodology described by Sauka et al. [9]. 

### 2.3. Insect Bioassays

Mixed spore-crystal suspensions of the strain exhibited insecticidal activity against *A. grandis* and no toxicity against *Alphitobius diaperinus* and *Cydia pomonella* (Table 2).

In consistency with both the lack of the PCR amplification of the *thuE* gene and the absence of its coding sequence in the Bt_UNVM-84 genome, no teratological effects were detected during the pupal emergence in adults of *Musca domestica* Linnaeus (Diptera: Muscidae).

## 3. Discussion

*B. thuringiensis* is the most used bacterium for controlling invertebrate pests, either through the use of spray formulations (e.g., Dipel, Xentary, etc.) or by expressing its insecticidal proteins in transgenic crops [22]. However, its effectiveness has been compromised, as some insect populations have evolved to become resistant through different mechanisms [23] to both spray formulations [24] and the most commonly used *B. thuringiensis* proteins (e.g., Cry1Ac) [25]. In addition to these problems, only a few types of insecticidal proteins from *B. thuringiensis* have been found to be effective against coleopteran pests [10,26], including those capable of controlling the cotton boll weevil *A. grandis*. This species is one of the most important pests, causing significant economic losses in the cotton industry in the Americas [26]. While chemical insecticides are efficient in controlling this pest in cotton, they are harmful to non-target organism, polluting the environment and increasing farmers’ expenses during the growing seasons [26]. In addition, the life cycle and behavior of this insect may limit its contact with synthetic insecticides, thereby enhancing survival and causing damage to cotton plants in cultivated areas. For these reasons, finding novel genes with insecticidal activity against coleopteran pests is crucial for improving biological control strategies in integrated pest management programs through the construction of genetically modified cotton with insect resistance against *A. grandis*. 

Here, we report the molecular and insecticidal characterization of a novel *Bacillus* sp. strain isolated from a soil sample obtained at Oncativo, Córdoba (Argentina). This strain exhibited ovoid to amorphous parasporal crystals, briefly described in a previous work [27]. These crystal shapes bear resemblance to those produced by strain INTA Fr7-4 and other strains showing insecticidal activity against *A. grandis* [14]. Moreover, similar parasporal-crystal shapes were identified through the cloning and expression of the Cry8Qa2 gene from strain INTA Fr7-4 into an acrystalliferous *B. thuringiensis* strain [28]. However, spore-crystal mixtures from this recombinant strain were not tested against *A. grandis*, and the mortality rate against *Anticarsia gemmatalis* Hübner (Lepidoptera: Noctuidae) was only 13.8% [28].

The genome sequence from strain Bt-UNVM-84 harbors seven insecticidal-like CDSs, showing similarity to well-known insecticidal proteins, including two pairs of binary Vpb1/Vpa2 proteins plus three Cry proteins showing similarity to Cry8 proteins (Table 1). Analysis by reads mapping has shown that the insecticidal CDSs are highly conserved with those already described in plasmid pFR206 [21]. Strain Bt_UNVM-84 also encodes CDSs showing similarity to the Mpp4Aa1, Mpp46Ab1, and Xpp22Ba1 proteins, with the last one absent in strain INTA Fr7-4, which has been described to exhibit insecticidal activity against *A. grandis* and *D. virgifera virgifera* [29]. Furthermore, Bt_UNVM-84 lacks the genes for toxins Cry1Ba and the binary Mpp23Aa/Xpp37Aa, which are highly active against *A. grandis* [26]. Although the novel strain was more closely related to *B. cereus* ATCC 14579 strain, *B. thuringiensis* is only differentiated from *B. cereus* by the production of typical parasporal crystal proteins [30] and was, therefore, designated here as *Bacillus thuringiensis* strain Bt_UNVM-84 instead of *B. cereus* sensu stricto biovar Thuringiensis by following the taxonomic nomenclature proposal for the *Bacillus cereus* group [31].

Insect bioassays using spore-crystal mixtures showed an interesting insecticidal activity, resulting in a 91% mean mortality for *A. grandis.* This suggests that the parasporal crystals may contain an active protein or proteins to control this pest. However, no toxicity has been observed against *C. pomonella* and *A. diaperinus*. The closely related INTA Fr7-4 strain has also been described to exhibit insecticidal activity against *A. grandis*, showing a lower percentage of mean mortality (32.5%). We hypothesize that this difference could be due to either the minor differences found in the coding nucleotide sequences (Table 1), which may be producing more active proteins, or the more efficient expression of some of them in the Bt_UNVM-84 strain. The results of this work suggest, at least at this stage, an exclusive coleoptericidal activity of strain Bt_UNVM-84. The identification of proteins in the crystals using liquid chromatography coupled with mass spectrometry should shed light on crystal composition and their role in the demonstrated activity against *A. grandis*. In addition, the absence of *thuE* coding sequences, confirmed by reads mapping, PCR amplification, and bioassays with *M. domestica*, suggests that this strain should be tested for the development of a sprayable formulation against other coleopteran pests. However, more experiments are necessary to elucidate which encoded gene or genes are responsible for the toxic activity against *A. grandis* and to unravel the full insecticidal potential of this novel and interesting *B. thuringiensis* strain. 

## 4. Conclusions

A novel *Bacillus* sp. strain showing insecticidal activity against *A. grandis* was isolated from Oncativo, Argentina, designated as *B. thuringiensis* strain Bt_UNVM-84 and characterized at the molecular and insecticidal levels. The genomic sequence exhibited a set of genes showing significant similarity with several insecticidal proteins active against coleopteran and lepidopteran pests. Strain INTA Fr7-4 was closely related to strains showing the same insecticidal-gene configuration but lower insecticidal activity against *A. grandis*. Although more studies are necessary to describe the full insecticidal potential of the strain and its encoded proteins, the results obtained in this work indicate that strain Bt_UNVM-84 is an interesting candidate to provide novel tools for the biological control of, at least, the cotton boll weevil *A. grandis.*

## 5. Materials and Methods

### 5.1. Strain Isolation and Characterization 

Soil samples were collected using a soil sampling tube from Establecimiento Norma Lucía farm in Oncativo, Córdoba province (Argentina), where *Medicago sativa* L. was planted. The final sample was a composite of five random sub-samples, totaling approximately 200 g of soil. This sample was stored at 4 °C in zip-lock bags until processed for bacteria isolation. Bacterial colonies, originating from sporulated species, were obtained as previously described by Iriarte et al. (1998) [32]. Colonies with a flat, dry, matt-white color and uneven borders (*B. thuringiensis*-like phenotype) were analyzed through PCR using degenerate primers (forward 5′-TATGCWCAAGCWGCCAATYTWCATYT-3′ and reverse 5′-GGRATAAATTCAATTYKRTCWA-3′) for the detection of three-domain *cry* genes [16]. In order to detect type I β-exotoxin production from strain Bt_UNVM-84, a qualitative PCR-based method for the detection of the *thuE* gene was performed as described previously by Sauka et al. [9] with the following forward 5′-GCGGCAGCCGTTTATTCAAA-3′ and reverse 5′-CCCCTTCCCATGGAGAAACA-3′ BEF primers, which produce 406-bp amplicons. The presence of parasporal crystals was examined using a light microscope, following the methodology described by Ammons et al. (2002) [15]. The production of parasporal crystals and their morphology were later confirmed by Scanning Electron Microscopy (SEM) at the Comprehensive Center for Electron Microscopy (CIME-CONICET, Argentina). The purified (axenic) sporulated colony was then stored at our bacterial collection in 15% glycerol at −80 °C. The composition of parasporal crystals and trypsin activation was determined by SDS-PAGE, following the procedure described by Pérez et al. [4], with *B. thuringiensis* svar. *kurstaki* HD-1strain used as reference.

### 5.2. Genome Sequencing and Annotation

Total DNA containing chromosome and plasmids was obtained using the Wizard genomic DNA purification kit (Promega, Madison, WI, USA), following the manufacturer’s instructions for the purification of DNA from Gram-positive bacteria. DNA was electrophoresed in 1% agarose gels stained with SYBR Safe (ThermoFisher Scientific, Waltham, MA, USA) and quantified using a PICODROP PICO 100 μL spectrophotometer. The purified DNA was then utilized to construct a pooled Illumina library, which was sequenced at the Genomics Unit from the National Institute of Agricultural Technology (INTA, Argentina) by using high-throughput Illumina sequencing technology. 

The obtained Illumina (raw) reads were analyzed, and trimmed regions were deleted before being assembled into contigs using Velvet plug in Geneious version R11 software suite (www.geneious.com, accessed on 10 November 2023), with the de novo assembly tool and default parameters. The resulting contigs were then analyzed with BLAST [20] using a customized personal non-redundant insecticidal protein database (2023 update). Genome annotation was initially performed with the NCBI Prokaryotic Genome Annotation Pipeline (2023 release) and the RAST server [33]. Species delimitation was performed with the Type (Strain) Genome Server [19]. The analysis of % pairwise identity by reads mapping over pFR260 plasmid and the genome of strain INTA Fr7-4 was performed using the *map to reference* tool embodied in Geneious R11 software. 

### 5.3. Insect Bioassays

The insecticidal activity of the strain was qualitatively evaluated against three insect species, including second instars of *Alphitobius diaperinus* Panzer (Coleoptera: Tenebrionidae), neonates of *Anthonomus grandis* Boheman (Coleoptera: Curculionidae), and neonates of *Cydia pomonella* Linnaeus (Lepidoptera: Tortricidae). Coleopteran and lepidopteran larvae were obtained from colonies reared at the Institute of Microbiology and Agricultural Zoology (IMYZA-INTA, Argentina).

For insect bioassays, strain Bt_UNVM-84 was grown in 100 mL of BM medium (2.5 g NaCl, 1 g KH_2_PO_4_, 2.5 g K_2_HPO_4_, 0.25 g MgSO_4_·7H_2_O, 0.1 g MnSO_4_·H_2_O, 5 g glucose, 2.5 g starch and 4 g yeast extract for 1 litter and with the pH adjusted to 7.2) at 340 rpm and 30 °C during approximately 72 h until complete autolysis was observed under the microscope. Spore-crystal mixtures were obtained by centrifugation at 12,000× *g* and 4 °C for 15 min; then, the pellets were freeze-dried, and the resultant powder composed of spore and crystals was kept at −20 °C until use. Each spore crystal mixture (final concentration of 5–1000 μg/mL) was incorporated into polypropylene conical tubes containing the corresponding artificial diet for each species (maintained at 60 °C) and poured into each well of a 24-well plate (Nunc 143982) [14]. Twenty-four coleopteran and lepidopteran larvae were used, and the bioassays were repeated twice. Mortality was recorded after 15 days at 29 °C for *A. diaperinus* and *A. grandis*, whereas 5 days were spent for *C. pomonella* at 29 °C. Larvae were accounted as dead if they did not respond to gentle probing. Distilled (sterile) water was used instead of spore crystal mixtures as mortality controls. Schneider-Orelli’s formula was used to calculate corrected mortality compared to the untreated control. The InfoStat software (Universidad Nacional de Córdoba, version 2014 was used for the statistical analysis, and the statistical significance was set at *p* < 0.05.

Strain Bt_UNVM-84 was also analyzed for its capability to synthesize type I β-exotoxin by counting the number of emerged *M. domestica* adults, following the methodology described by Sauka et al. [9] and using the β-exotoxin producer strain HD-2 as positive control.

## Figures and Tables

**Table 1:** Insecticidal CDSs comparison by mapping Bt_UNVM-84 reads over pFR260 megaplasmid.

CDSs ^a^	% Pairwise Nucleotide Identity	Contig	% Ref-Seq Coverage	Gene Length (bp)
*vpa2Ah1*	98.8	NCA	93.9	1338
*vpb1Ea1*	99.0	52	99.7	2625
*vpa2Ah2*	98.9	61	100	1338
*vpb1Ea2*	98.7	61	100	2619
*cry8Kb3*	96.3	61	42.5	3510
*cry8Pa3*	97.7	39	52.8	3531
*cry8Qa2*	97.7	75	98.7	3555

^a^ Closest homolog. NCA = no contig assigned, detected by read-mapping over plasmid pFR206 [21].

**Table 2:** Bioassays with insects were conducted with the whole strain (spore-crystal mixtures).

Species	% Average Mortality ± SD	% Corrected Mortality ± SD
*C. pomonella*	25.0 ± 5.9 ^a^	5.3 ± 7.5
*A. diaperinus*	21.7 ± 12.3 ^b^	7.2 ± 14.6
*A. grandis*	91.7 ± 5.9 ^b^	91.1 ± 6.3

^a^ 5 μg/mL diet. ^b^ 1 mg/mL diet.

## Data Availability

This whole-genome shotgun project has been deposited in DDBJ/ENA/GenBank under the accession number JAWUAH000000000. The version described in this paper is the first version, JAWUAH010000000. Illumina paired-end (raw) reads were deposited at Sequence Read Archive (SRA) database under accession PRJNA1035773.

## References

[1] Melo A.L.d.A., Soccol V.T., Soccol C.R. (2016). *Bacillus thuringiensis*: Mechanism of action, resistance, and new applications: A review. Crit. Rev. Biotechnol..

[2] Kumar S., Chandra A., Pandey K.C. (2008). *Bacillus thuringiensis* (Bt) transgenic crop: An environment friendly insect-pest management strategy. J. Environ. Biol..

[3] Pathak V.M., Verma V.K., Rawat B.S., Kaur B., Babu N., Sharma A., Dewali S., Yadav M., Kumari R., Singh S. (2022). Current status of pesticide effects on environment, human health and it’s eco-friendly management as bioremediation: A comprehensive review. Front. Microbiol..

[4] Crickmore N., Berry C., Panneerselvam S., Mishra R., Connor T.R., Bonning B.C. (2021). A structure-based nomenclature for *Bacillus thuringiensis* and other bacteria-derived pesticidal proteins. J. Invertebr. Pathol..

[5] Chakroun M., Banyuls N., Bel Y., Escriche B., Ferré J. (2016). Bacterial Vegetative Insecticidal Proteins (Vip) from Entomopathogenic Bacteria. Microbiol. Mol. Biol. Rev..

[6] Yin Y., Flasinski S., Moar W., Bowen D., Chay C., Milligan J., Kouadio J.L., Pan A., Werner B., Buckman K. (2020). A new *Bacillus thuringiensis* protein for Western corn rootworm control. PLoS ONE.

[7] Schnepf E., Crickmore N., Van Rie J., Lereclus D., Baum J., Feitelson J., Zeigler D.R., Dean D.H. (1998). *Bacillus thuringiensis* and Its Pesticidal Crystal Proteins. Microbiol. Mol. Biol. Rev..

[8] Liu X., Ruan L., Peng D., Li L., Sun M., Yu Z. (2014). Thuringiensin: A thermostable secondary metabolite from *Bacillus thuringiensis* with insecticidal activity against a wide range of insects. Toxins.

[9] Sauka D.H., Pérez M.P., López N.N., Onco M.I., Berretta M.F., Benintende G.B. (2014). PCR-based prediction of type I β-exotoxin production in *Bacillus thuringiensis* strains. J. Invertebr. Pathol..

[10] Dominguez-Arrizabalaga M., Villanueva M., Fernandez A.B., Caballero P. (2019). A Strain of *Bacillus thuringiensis* Containing a Novel *cry7Aa2* Gene that Is Toxic to *Leptinotarsa decemlineata* (Say) (Coleoptera: Chrysomelidae). Insects.

[11] Sánchez-Reyes U.J., Jones R.W., Raszick T.J., Ruiz-Arce R., Sword G.A. (2022). Potential Distribution of Wild Host Plants of the Boll Weevil (*Anthonomus grandis*) in the United States and Mexico. Insects.

[12] Health E.P.o.P., Jeger M., Bragard C., Caffier D., Candresse T., Chatzivassiliou E., Dehnen-Schmutz K., Gilioli G., Gregoire J.C., Jaques Miret J.A. (2017). Pest categorisation of *Anthonomus grandis*. EFSA J..

[13] Rolim G.G., Coelho R.R., Antonino J.D., Arruda L.S., Rodrigues A.S., Barros E.M., Torres J.B. (2021). Field-evolved resistance to beta-cyfluthrin in the boll weevil: Detection and characterization. Pest. Manag. Sci..

[14] Pérez M.P., Sauka D.H., Onco M.I., Berretta M.F., Benintende G.B. (2017). Selection of *Bacillus thuringiensis* strains toxic to cotton boll weevil (*Anthonomus grandis*, Coleoptera: Curculionidae) larvae. Rev. Argent. Microbiol..

[15] Ammons D., Rampersad J., Khan A. (2002). Usefulness of staining parasporal bodies when screening for *Bacillus thuringiensis*. J. Invertebr. Pathol..

[16] Noguera P.A., Ibarra J.E. (2010). Detection of new *cry* genes of *Bacillus thuringiensis* by use of a novel PCR primer system. Appl. Environ. Microbiol..

[17] Fang Y., Li Z., Liu J., Shu C., Wang X., Zhang X., Yu X., Zhao D., Liu G., Hu S. (2011). A pangenomic study of *Bacillus thuringiensis*. J. Genet. Genom..

[18] Ibrahim M.A., Griko N., Junker M., Bulla L.A. (2010). Bacillus thuringiensis. Bioeng. Bugs..

[19] Meier-Kolthoff J.P., Göker M. (2019). TYGS is an automated high-throughput platform for state-of-the-art genome-based taxonomy. Nat. Commun..

[20] Altschul S.F., Gish W., Miller W., Myers E.W., Lipman D.J. (1990). Basic local alignment search tool. J. Mol. Biol..

[21] Navas L.E., Amadio A.F., Ortiz E.M., Sauka D.H., Benintende G.B., Berretta M.F., Zandomeni R.O. (2017). Complete Sequence and Organization of pFR260, the *Bacillus thuringiensis* INTA Fr7-4 Plasmid Harboring Insecticidal Genes. J. Mol. Microbiol. Biotechnol..

[22] Romeis J., Naranjo S.E., Meissle M., Shelton A.M. (2019). Genetically engineered crops help support conservation biological control. Biol. Control.

[23] Jurat-Fuentes J.L., Heckel D.G., Ferré J. (2021). Mechanisms of Resistance to Insecticidal Proteins from *Bacillus thuringiensis*. Annu. Rev. Entomol..

[24] Zago H.B., Siqueira H., Pereira E.J., Picanço M.C., Barros R. (2014). Resistance and behavioural response of *Plutella xylostella* (Lepidoptera: Plutellidae) populations to *Bacillus thuringiensis* formulations. Pest. Manag. Sci..

[25] Liu Z., Fu S., Ma X., Baxter S.W., Vasseur L., Xiong L., Huang Y., Yang G., You S., You M. (2020). Resistance to *Bacillus thuringiensis* Cry1Ac toxin requires mutations in two *Plutella xylostella* ATP-binding cassette transporter paralogs. PLoS Pathog..

[26] De Oliveira J.A., Negri B.F., Hernández-Martínez P., Basso M.F., Escriche B. (2023). Mpp23Aa/Xpp37Aa Insecticidal Proteins from *Bacillus thuringiensis* (Bacillales: Bacillaceae) Are Highly Toxic to *Anthonomus grandis* (Coleoptera: Curculionidae) Larvae. Toxins.

[27] Peralta C., Sauka D.H., Marozzi A., Del Valle E.E., Palma L. (2021). Argentinean *Bacillus thuringiensis* strains exhibiting distinct morphology of their parasporal crystals. Rev. Argent. Microbiol..

[28] Amadio A.F., Navas L.E., Sauka D.H., Berretta M.F., Benintende G.B., Zandomeni R.O. (2013). Identification, cloning and expression of an insecticide *cry8* gene from *Bacillus thuringiensis* INTA Fr7-4. J. Mol. Microbiol. Biotechnol..

[29] Isaac B., Krieger E.K., Light A.-M., Farhad M., Sivasupramanian S. (2001). Polypeptide Compositions Toxic to Anthonomus Insects, and Methods of Use. U.S. Patent.

[30] Helgason E., Okstad O.A., Caugant D.A., Johansen H.A., Fouet A., Mock M., Hegna I., Kolstø A.B. (2000). *Bacillus anthracis*, *Bacillus cereus*, and *Bacillus thuringiensis*—One species on the basis of genetic evidence. Appl. Environ. Microbiol..

[31] Carroll L.M., Wiedmann M., Kovac J. (2020). Proposal of a Taxonomic Nomenclature for the *Bacillus cereus* Group Which Reconciles Genomic Definitions of Bacterial Species with Clinical and Industrial Phenotypes. mBio.

[32] Iriarte J., Bel Y., Ferrandis M.D., Andrew R., Murillo J., Ferré J., Caballero P. (1998). Environmental distribution and diversity of *Bacillus thuringiensis* in Spain. Syst. Appl. Microbiol..

[33] Aziz R.K., Bartels D., Best A.A., DeJongh M., Disz T., Edwards R.A., Formsma K., Gerdes S., Glass E.M., Kubal M. (2008). The RAST Server: Rapid annotations using subsystems technology. BMC Genom..

